# Ongoing Oxidative Stress Causes Subclinical Neuronal Dysfunction in the Recovery Phase of EAE

**DOI:** 10.3389/fimmu.2016.00092

**Published:** 2016-03-14

**Authors:** Helena Radbruch, Daniel Bremer, Robert Guenther, Zoltan Cseresnyes, Randall Lindquist, Anja E. Hauser, Raluca Niesner

**Affiliations:** ^1^Department of Neuropathology, Charité – Universitätsmedizin Berlin, Berlin, Germany; ^2^German Rheumatism Research Center (DRFZ) a Leibniz Institute, Berlin, Germany; ^3^Immundynamics, Charité – Universiätsmedizin Berlin, Berlin, Germany

**Keywords:** NOX, EAE/MS, intravital imaging, FLIM–FRET, calcium

## Abstract

Most multiple sclerosis (MS) patients develop over time a secondary progressive disease course, characterized histologically by axonal loss and atrophy. In early phases of the disease, focal inflammatory demyelination leads to functional impairment, but the mechanism of chronic progression in MS is still under debate. Reactive oxygen species generated by invading and resident central nervous system (CNS) macrophages have been implicated in mediating demyelination and axonal damage, but demyelination and neurodegeneration proceed even in the absence of obvious immune cell infiltration, during clinical recovery in chronic MS. Here, we employ intravital NAD(P)H fluorescence lifetime imaging to detect functional NADPH oxidases (NOX1–4, DUOX1, 2) and, thus, to identify the cellular source of oxidative stress in the CNS of mice affected by experimental autoimmune encephalomyelitis (EAE) in the remission phase of the disease. This directly affects neuronal function *in vivo*, as monitored by cellular calcium levels using intravital FRET–FLIM, providing a possible mechanism of disease progression in MS.

## Introduction

Multiple sclerosis (MS) is a chronic neuroinflammatory disease, with most patients exhibiting a relapsing and remitting course of disease. The neurological damage is a consequence of a mainly T cell-driven immune reaction against myelin in the central nervous system (CNS) (1). Macrophages/microglia, B, and T cells create an acute inflammatory setting, resulting in demyelination and neuronal damage. Most of the patients who experience a second episode develop further relapses. Despite the intensive analysis of the acute immune attack, only little is known about the processes going on at the lesion site after the initial insult (2, 3).

Why and where do new relapses appear? What factors determine the chronicity of a lesion and the course of disease? “Old” lesions appear morphologically inert and are characterized by single perivascular T cells, minimal axonal damage in histological stainings with anti-amyloid precursor protein (APP) antibodies and a dominant fibrotic glial scar (4, 5). In contrast to the progressive disease phase, the inflammatory phase is well modeled by murine experimental autoimmune encephalomyelitis (EAE). In this mouse model using an immunization with MOG_35–55_ peptide, acute clinical signs remit after a few days and mice enter into a chronic phase with or without a residuum of neurological deficits (1). Reactive oxygen species (ROS) generated by invading and resident CNS macrophages have been implicated in mediating demyelination and axonal damage (6–8). In this study, we address implications of the ceased immune attack for the CNS tissue, beyond the fact that the majority of peripheral immune cells disappeared. In MS patients, we previously detected an ongoing over-activation of NADPH oxidases (especially NOX2) in blood monocytes during remission (8). Using intravital NAD(P)H fluorescence lifetime imaging in mice affected by EAE, during the inflammatory phase (onset and peak of the disease), we detected a massively increased amount of functional NADPH oxidases (NOX1–4, DUOX1, 2) within the CNS as compared to healthy controls (8). Using the same method, we investigated whether functional NADPH oxidases are still present in the CNS after recovery of EAE and, thus, whether oxidative stress is still ongoing in absence of peripheral infiltration of the CNS. We simultaneously monitor calcium concentrations in neurons using intravital FRET–FLIM-based neuronal calcium imaging to evaluate the reaction of the neurons on the altered CNS environment over the course of EAE development and remission. Thereby, we investigate whether in mice with clinical recovery morphologically inert appearing lesions exhibit residual inflammation, as reflected by increased oxidative stress and sub-clinical neuronal dysfunction, in order to better understand mechanisms of chronicity and disease progression in MS and related diseases.

## Results

### Characterization of the Remission Phase in the CNS of Mice Affected by EAE

The grade of inflammation in brain stem of mice with EAE after clinical recovery (remission) was characterized and compared to animals at the peak of disease and to healthy controls. Our aim was to first characterize peripheral and CNS resident cellular compartments during the remission phase by means of intravital imaging and to corroborate previous results concerning the lack of overt inflammation in this phase.

### Characterization of Cellular Markers in the CNS, during EAE Remission

**Figure 1:**
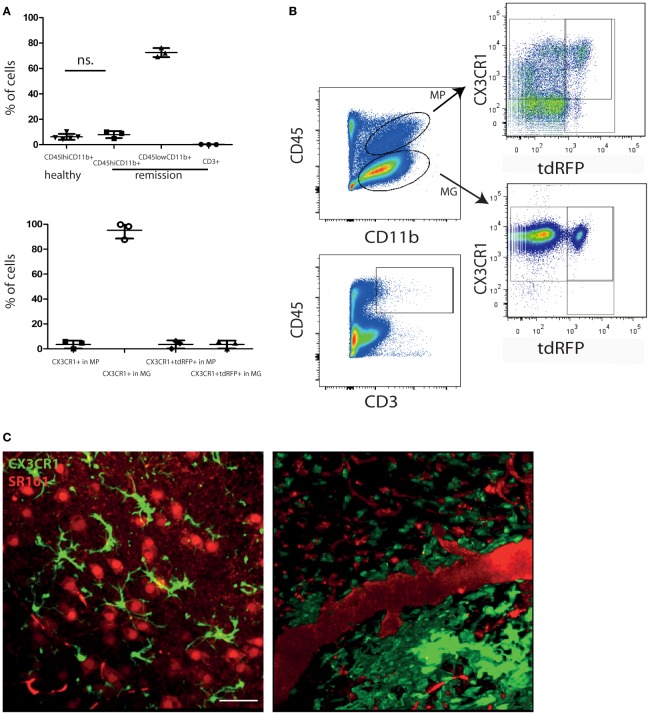
**Peripheral immune infiltration of the CNS has largely resolved in the remission phase of EAE**. **(A)** FACS analysis of CNS cells after recovery of EAE shows a low immune infiltrate with few monocytes/macrophages (CD45^high^CD11b^+^; MP) and CD3^+^ cells. Most of the CD45 expressing cells are CD45^low^CD11b^+^ (microglia; MG). CD45^high^CD11b^+^ frequencies are comparable to healthy untreated mice (*n* = 5; ±SD). We applied an unpaired *t*-test to statistically evaluate the results. In the CD45^high^CD11b^+^ fraction (MP), only few cells express CX3CR1 but most of the cells in the CD45^low^CD11b^+^ fraction (MG). The overlap of CX3CR1 and tdRFP was comparable in both cell types around 3% (*n* = 3; ±SD, clinical information listed in Table 1) **(B)** Exemplarily gating strategy of the FACS analysis of whole CNS in mice after recovery of EAE. **(C)** Projection of 3D intravital fluorescence image in the brain stem of a *CX3CR1*^+/−^ *EGFP* mouse in health and during the remission phase of EAE. The astrocytes (and blood vessels) are labeled by i.v. injected SR101 (red), while the microglia are expressing EGFP (green). Scale bar = 50 μm. The colocalization of the EGFP and SR101 signals, i.e., overlap of the microglial and astrocytic markers, respectively, amounts to 3.6 ± 1.8%.

It is widely accepted that in MS, inactive CNS lesions with no signs of immune infiltration are detectable. In our EAE model, some mice show a complete clinical recovery of EAE signs. We characterized these mice by FACS analysis of whole murine CNS (brain and spinal cord) and demonstrated that both monocytes/macrophages (CD45^high^CD11b^+^ cells) and T cells (CD45^high^CD3^+^ cells) disappear from the CNS during the remission phase of EAE. Only 7.9 ± 2.8% of the isolated CNS cells were CD45^high^CD11b^+^ cells (macrophages/monocytes), comparable with healthy controls with 6.2 ± 2.4% (Figures 1A,B), whereas their frequency during onset and peak of EAE was previously shown to be strongly increased, to ~50% of the infiltrates (8–10). The majority of cells after EAE recovery were cells with characteristics of microglia: 72.5 ± 3.6% were CD45^low^CD11b^+^ of which 95.2 ± 6.7% expressed *CX3CR1*. The overlap of *CX3CR1* and tdRFP (LysM) was in both compartments under 5% (3.5 ± 3.2% for CD45^high^CD11b^+^ cells and 3.5 ± 3.1% for CD45^low^CD11b^+^ cells). CD45^high^CD3^+^ cells – typically present during the peak of EAE (11) – mainly disappeared after EAE recovery, constituting only 0.2 ± 0.1% of total cell number. All these findings are in line with the low clinical scores of the mice (between 0 and 0.5; Table 1) and are consistent with previous observations of cellular compositions after EAE recovery (10). Our results encompass two independent EAE experiments with a total number of *n* = 3 mice analyzed in remission phase (Table 1) and *n* = 5 analyzed healthy mice.

**Table 1:** **Mouse strains, EAE data of the mice and mean NOX activation area values within lesions or gliosis/astrogliosis areas with SD per animal (6–20 areas within the brain stem per animal)**.

EAE ID	Mouse strain	EAE score at analysis time point	Maximum EAE score	Mean NOX activation area (%)	SD
1	CX3CR1.EGFP	1.5	1.5	17.88	5.13
1	CerTN L15 × LysM tdRFP	1.0	1.0	13.20	1.06
1	CerTN L15 × LysM tdRFP	0.5	2.0	8.15	2.25
2	CerTN L15 × LysM tdRFP	2.5	2.5	16.99	9.02
2	CerTN L15 × LysM tdRFP	0.5	1.5	9.91	3.97
3	CerTN L15 × LysM tdRFP	2.0	2.0	10.65	0.51
3	CerTN L15 × LysM tdRFP	2.0	2.0	7.18	1.42
3	CX3CR1.EGFP	1.5	1.5	11.61	1.34
3	CX3CR1.EGFP	0.0	1.5	9.27	3.25
3	CX3CR1.EGFP	0.0	1.0	8.77	3.97
4	CX3CR1.EGFP	0.0	2.0	11.53	5.38
4	CX3CR1.EGFP	0.0	1.5	9.71	2.79
4	CX3CR1.EGFP	0.5	2.0	12.19	4.65
4	CerTN L15 × LysM tdRFP	0.0	2.0	–	–
5	CerTN L15 × LysM tdRFP	0.5	3.5	–	–
5	CerTN L15 × LysM tdRFP	0.5	3.5	–	–

**Healthy controls**	**Mouse strain**			**Mean NOX activation area (%)**	**SD**

1	CerTN L15 × LysM tdRFP			0.37	0.13
2	C57BL/6			2.84	0.29
3	CerTN L15 × LysM tdRFP			0.47	0.09
4	C57BL/6			0.60	0.28
5	CerTN L15 × LysM tdRFP			2.08	0.91

*We included five independent EAE experiments and five healthy controls for the intravital NAD(P)H–FLIM experiments*.

Using intravital microscopy in *CX3CR1*^+/−^*EGFP* mice (*n* = 3) after EAE recovery, we could show a reduced overlap of 3.6 ± 1.8% between EGFP and i.v. injected sulforhodamin 101 (SR101), which labels astrocytes both in health and in peak EAE (8). These results are similar to the overlap measured in healthy *CX3CR1*^+/−^*EGFP* mice labeled by i.v. injection with SR101 (2.7 ± 1.1% overlap, Figure 1C).

### Intravital Imaging Reveals Morphologic Features of EAE Remission in the CNS

We performed intravital imaging experiments in the brain stem of *CerTN L15* × *LysM tdRFP* mice (neurons express the Ca^2+^ indicator TN L15, while predominantly *LysM*^+^ phagocytes express tdRFP) and of *CX3CR1*^+/−^ *EGFP* mice (microglia/macrophages express EGFP, while predominantly astrocytes are labeled by SR101).

**Figure 2:**
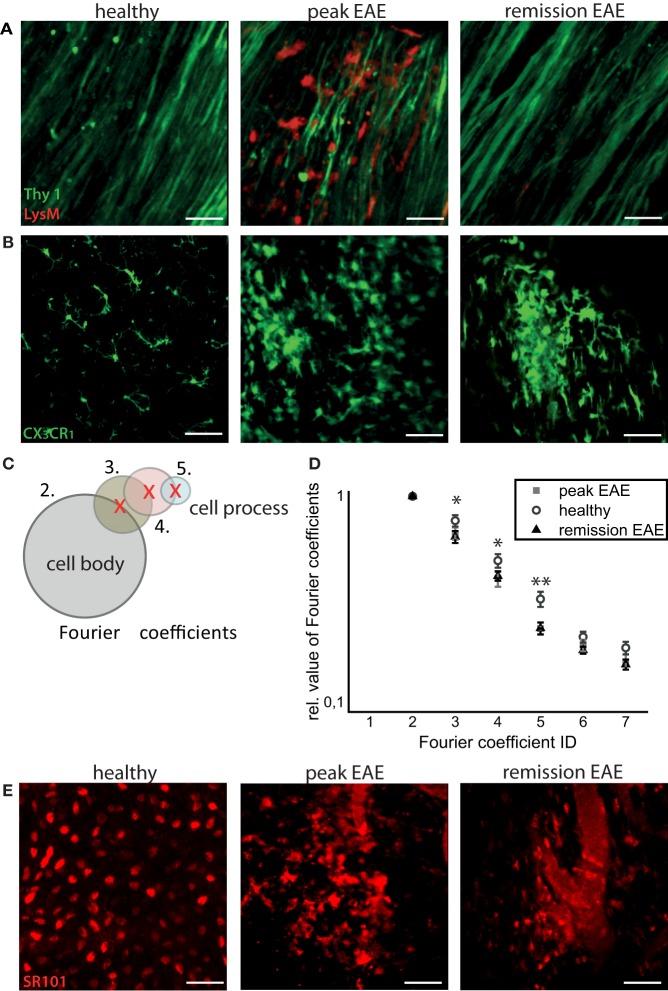
**Intravital imaging reveals that remission in EAE correlates with lack of overt immune infiltration, with persisting disruptions of the microglial and astrocytic networks**. **(A)** 3D intravital fluorescence images in the brain stem of *CerTN L15* × *LysM tdRFP* mice in health (*n* = 5), at peak EAE (*n* = 6) and in the remission phase (*n* = 2). λ_exc_ = 850 + 1110 nm, λ_em_ = 525 ± 25 nm (Thy1-Citrine in neurons depicted in green), λ_em_ = 593 ± 20 nm (LysM tdRFP in phagocytes depicted in red), scale bar = 50 μm. **(B)** 3D intravital fluorescence images in the brain stem of *CX3CR1*^+/−^ *EGFP* mice in health (*n* = 3), at peak EAE (*n* = 4) and in the remission phase (*n* = 5). λ_exc_ = 935 nm, λ_em_ = 525 ± 25 nm (CX*3*CR*1*^+/−^ EGFP in microglia/macrophages depicted in green), scale bar = 50 μm. **(C)** Using higher-order Fourier coefficients, we describe the complex shape of microglia. The first Fourier coefficient describes the position of the cells, the second coefficient the sphericity of the cell body and starting from the third Fourier coefficient, the ramification of all cell processes is reproduced: the higher the values of high-order Fourier coefficients with respect to the second Fourier coefficient, the higher the degree of ramification and length of cellular processes of microglia. **(D)** The different shapes of the microglia, shown in **(B)**, were classified in health (71 cells) at peak EAE (57 cells) and in its remission phase (63 cells). The difference between the values of the third, fourth, and fifth Fourier coefficients is significant between healthy controls and remission, but not significant between peak of EAE and remission of EAE. Statistical significance was determined by ANOVA (*p < 0.05, **p < 0.01, ***p < 0.001). **(E)** Projection of 3D intravital fluorescence images in the brain stem of C57/B6 mice i.v. injected with sulforhodamine 101 (SR101) in health (*n* = 2), at peak EAE (*n* = 4) and in the remission phase (*n* = 3). λ_exc_ = 880 nm, λ_em_ = 593 ± 20 nm (SR101 in astrocytes depicted in red), scale bar = 50 μm.

Infiltration of the CNS by *LysM*^+^ cells is transient, and varies with the stage of disease. In health, practically no *LysM*^+^ cells are present except for few perivascular *LysM*^+^ microglia. During peak of EAE, many *LysM*^+^ cells are present within CNS lesions, and they mostly disappear during the remission phase. We could only identify isolated regions where *LysM*^+^ cells were present inside or in the close proximity to blood vessels or meninges (Figure 2A).

In contrast to the peripheral immune cells, the inflammatory-induced gliosis of CNS-resident cells [microglia and astrocytes having phagocytic capacity (8)] persists after EAE recovery (Figure 2B). We evaluated shape and function of astrocytes and microglia to test our hypothesis that the function of these CNS cells, in chronic neuroinflammation, has persistently (pathologically) elevated phagocytic features, even in the absence of peripheral immune cells.

First, we quantitatively analyzed the shape of microglia, based on the fact that resting microglia, typical for healthy CNS, are highly ramified, whereas activated microglia, especially those having a phagocytic function, lose their cellular processes and adopt an amoeboid shape. The amoeboid shapes are expected to be found especially in the diseased CNS (10).

We used Fourier coefficients to quantify and reproduce the ramified shape of microglia and to quantify their shape changes in the remission phase as compared to health and peak of the disease. Briefly, single microglia cells were segmented from intravital microscopy data acquired in the brain stem of healthy and EAE mice (in peak and remission phase). Six two-dimensional projections from each three-dimensional object (cell) were generated, and their shape was approximated by overlapping circles as displayed in Figure 2C. Each layer of circles is mathematically characterized by a scalar parameter called Fourier coefficient. Thus, the first Fourier coefficient defines the position of a cell, the second defines its dimensions by approximating it with a perfect sphere, and the next Fourier coefficients define the number and length of cellular processes. Each cellular process is approximated by a set of spheres of various diameters, with the center on the surface of the most distant, previous sphere (Figure 2C). The higher the ramification and the length of cellular processes, the larger are the relative values of the high-order Fourier coefficients with respect to the second Fourier coefficient. We found a high shape similarity of microglia during the remission phase (118 cells) and of those imaged at the lesion site, at the peak of the disease (57 cells). In comparison to resting microglia in healthy controls (71 cells), the similarity was rather low (Figure 2D). The third, fourth, and fifth Fourier coefficients show a significant difference (using an ANOVA test) both in remission and in peak as compared to healthy controls. The results encompass two independent EAE experiments with *n* = 3 healthy controls, *n* = 2 mice at peak EAE, and *n* = 4 mice during the remission phase. The findings of our intravital experiments demonstrate that in remission, after clinical recovery, microglia retain an activated morphology, suggesting that their function remains predominantly phagocytic despite the fact that clinical symptoms disappeared.

Consistent with the results of shape analysis of microglia, the astrocytic network appears intact in healthy controls (*n* = 2), whereas during peak EAE (*n* = 4) and the remission phase (*n* = 3), it appears disrupted (Figure 2E). Additionally, the fine astrocytic processes completely disappear and are replaced by thick perivascular processes, while the astrocytic cell bodies adopt ameboid shapes (Figure 2E). A quantification of these observations is rather difficult. Even if a good segmentation of the single astrocytes and their processes is given, currently there is no available mathematical approach or set of mathematical parameters to summarize the complexity of the profound changes of the astrocytic network. However, altogether the observations regarding morphological modifications suggest that the astrocytes are also shifted toward a phagocytic function.

### Subclinical Neuronal Dysfunction Correlates with Oxidative Stress without Overt Immune Infiltration after Recovery of EAE

Altered morphology often indicates functional changes, but morphology is not a direct measure of the cellular function. To evaluate alterations in cellular function over the course of EAE, we used intravital NAD(P)H fluorescence lifetime imaging (FLIM), as previously described (8), to detect the over-activation of NADPH oxidases (NOX1–4, DUOX1, 2). As we previously showed in intravital imaging experiments of mice affected by EAE, a concentration of ~200 μM of ROS is detectable in the brain stem, in EAE, using local ROS labeling with Amplex Red. In contrast, in healthy animals, we could not detect any ROS generation. As ROS molecules are highly reactive and diffusive, their detection is limited and the analysis of their cellular source practically impossible. We circumvent this disadvantage by detecting the catalyzer of ROS production, i.e., NOX enzymes, using NAD(P)H–FLIM *in vivo*. We previously showed that high ROS concentration in the brain stem of EAE animals correlates with the over-activation of NOX enzymes as detected by intravital NAD(P)H–FLIM (12). The fluorescence lifetime of NADPH bound to NADPH oxidases is ~3650 ps (12), differing from generally active NADH- and NADPH-dependent enzymes [fluorescence lifetime of NAD(P)H ~2200 ps]. The over-activation of NADPH oxidases is a prerequisite of oxidative stress – known to be one of the main causes of neuronal dysfunction in chronic neuroinflammation (6, 13).

**Figure 3:**
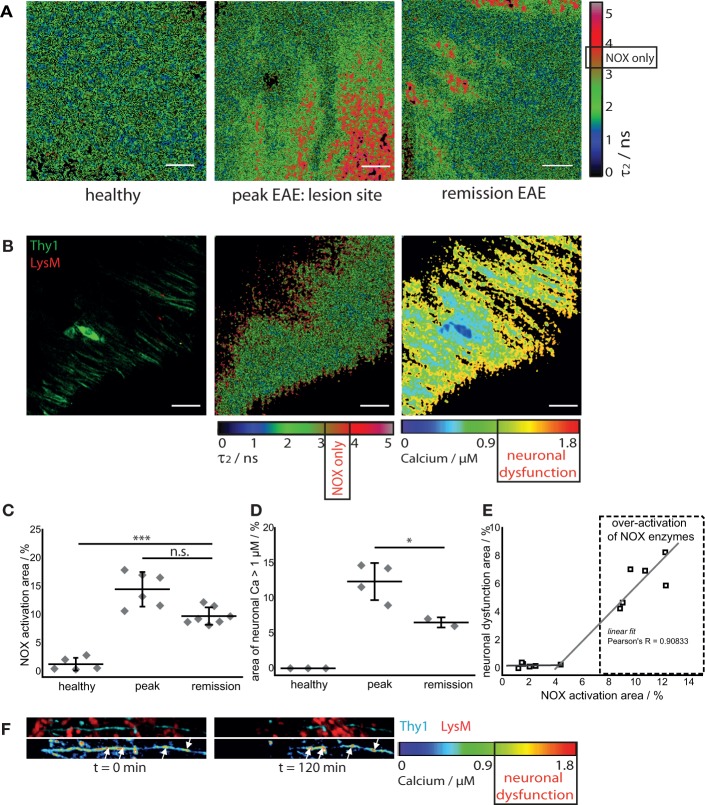
**NOX enzymes activation correlates with neuronal dysfunction in the remission phase of EAE**. **(A)** τ_2_ (enzyme-bound) NAD(P)H–FLIM images acquired in the brain stem of healthy mice (*n* = 5) and of mice affected by EAE at peak of the disease (*n* = 6) and in the remission phase (*n* = 7). The results encompass four independent EAE experiments. Scale bar = 30 μm. **(B)** Intravital fluorescence intensity image, NAD(P)H–FLIM image and FRET–FLIM neuronal calcium image acquired in the brain stem of a *CerTN L15* × *LysM tdRFP* mouse in the remission phase of EAE. Scale bar = 30 μm. **(C)** Quantification of the mean NOX activation area of individual mice at peak EAE (*n* = 6) and in the remission phase (*n* = 7), four independent EAE experiments. While the mean are of NOX activation is strongly increased in the remission phase of EAE as compared to healthy controls, we could observe only a slight decrease of the NOX activation area as compared to peak of EAE (at lesion sites). **(D)** Quantification of the neuronal dysfunction area characterized by a neuronal calcium concentration larger than 1 μM at peak EAE (*n* = 4) and in the remission phase (*n* = 2), two independent EAE experiments. The area of elevated neuronal calcium (area of neuronal dysfunction) is slightly reduced in the remission phase of EAE as compared to the peak of the disease. However, since in healthy controls there is no elevated neuronal calcium, in both phases of EAE, the elevated calcium indicates massive neuronal dysfunction. **(E)** Direct correlation between NOX enzymes over-activation area and neuronal dysfunction area in the remission phase of EAE, within the brain stem (*n* = 2 mice). All images are acquired at 30–150 μm depth within the brain stem (*z*-step = 2 μm). **(F)** Intravital 3D images of the brain stem of a *CerTN L15* × *tdRFP* mouse affected by EAE, at peak of the disease, at an arbitrary time point *t* = 0 and 120 min later. The upper panels show intensity images of axons (cyan, Thy1) and of immune cells (red), whereas the lower images show the corresponding FRET ratio images (Calcium images) of the axons. At the contact sites between axons and immune cells, we observe strongly increased neuronal calcium. After 2 h, at exactly these sites, we observed dramatic morphological changes of the axons, i.e., appearance of ovoid bodies and axonal disruption. The axonal disruption and ovoid bodies formation along the axon is indicated by white arrows in the lower panels of **(F)**. Statistical evaluation in **(C,D)** was determined by ANOVA tests (*p < 0.05, **p < 0.01, ***p < 0.001).

In healthy mice, intravital NAD(P)H–FLIM of the brain stem reveals predominantly metabolic enzyme activity (8). At peak of EAE, the lesion site is associated with vast areas of activated NADPH oxidases, leading to increased oxidative stress (8). Surprisingly, even if overt inflammation and the clinical symptoms disappear, a local activation of NADPH oxidases does not decline to levels found in healthy mice. While the area of NOX enzymes activation in the brain stem of healthy mice amounts in average to 1.8 ± 1.3%, the same average value at peak of the disease significantly increases eightfold to 15.6 ± 5.1% and declines only slightly to 9.4 ± 1% during the remission phase, still over fivefold higher than in healthy mice (Figures 3A,C).

To determine if this increased activation of NAD(P)H oxidases was associated with subclinical neuronal dysfunction, we determined the neuronal calcium level using intravital FRET–FLIM in the brain stem of *CerTN L15* × *LysM tdRFP* mice affected by EAE, as previously described (8, 14, 15). Associated with the persisting oxidative stress, we observed increased neuronal calcium indicating subclinical neuronal dysfunction, and progressing to neuronal damage, within the areas of elevated oxidative stress. The area of measured neuronal dysfunction in the remission phase (6.2 ± 1.7%) is lower than at peak of disease (11.7 ± 2.8%) (16), in line with higher scores during peak than in the recovery phase (Table 1); however, it is significantly higher than in healthy mice, in which no neuronal dysfunction can be observed using the same approach (Figures 3B,D) (14). As depicted in Figure 3E, in regions with no or very low over-activation of NOX enzymes (<4% of the total observed area), no neuronal dysfunction can be detected. Beyond this value, the oxidative stress regime is established and neuronal dysfunction linearly increases with increasing area of NOX enzymes activation, within the CNS (Pearson’s *R* = 0.90883).

As we and others previously demonstrated, a sustained elevated calcium concentration in neurons, both *in vivo* and in primary neuronal cultures, can precede morphological changes and finally neuronal death. In the brain stem of a *CerTN L15* × *LysM tdRFP* mouse affected by EAE, enhanced contact of axons with peripheral *LysM* cells correlate with an increased calcium baseline in axons (Figure 3F). Imaging over 2 h reveals after recovery of EAE sites of increased calcium concentration do not correlate with dramatic morphological changes such as ovoid bodies or even axonal disruption, in contrast to the situation in peak EAE as depicted in Figure 3F (white arrows). Since the *TN L15* genetically encoded biosensor reacts slowly to calcium (within few hundred milliseconds), it cannot track the physiologic calcium oscillations typical for neurons, but records only the low average baseline (≈100 nM) (14).

### Microglia and Astrocytes Mainly Contribute to Oxidative Stress after EAE Recovery

**Figure 4:**
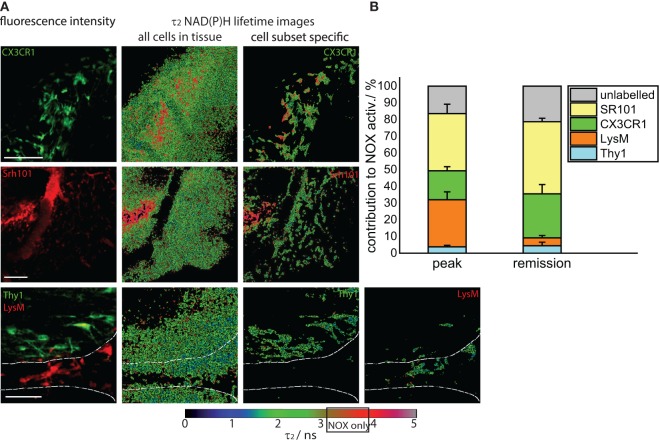
**Cellular origin of oxidative stress in the remission phase of EAE**. **(A)** Intravital fluorescence images and enzyme-bound NAD(P)H fluorescence lifetime images of all cells and of specific cell subtypes acquired in the brain stem of transgenic mice affected by EAE, in the remission phase. We evaluated *CerTN L15* × *LysM tdRFP* mice, in which neurons Thy1 express the calcium indicator TN L15 and LysM^+^ phagocytes express tdRFP, and *CX3CR1*^+/−^ *EGFP* mice, in which microglia/macrophages express EGFP. Astrocytes were intravitally labeled with sulforhodamine 101 by i.v. injection at least 2 h before imaging. The white lines in the fluorescence and fluorescence lifetime images at the bottom of **(A)** demarcate the outline of a blood vessel. Hence, it becomes obvious that most of *LysM*^+^ cells reside within the blood vessels, and only few can be found at the border to the CNS parenchyma. Scale bars = 50 μm. **(B)** Contribution of the individual cell subsets to the over-activation of the NOX enzymes in the CNS of mice during the remission phase of EAE (*n* = 2 CerTN L15 × LysM tdRFP mice – neurons/Thy1 and LysM; *n* = 5 CX3CR1^+/−^ EGFP mice – microglia/CX3CR1 cells; *n* = 3 mice labeled with sulforhodamine 101 – astrocytes). The results encompass four independent EAE experiments.

Next, we elucidated the specific cellular origin of the persistent oxidative stress in the CNS during the remission phase. The approach used in our study – performing endogenous NAD(P)H–FLIM in the CNS of mice with differently fluorescing cell subsets (*LysM*^+^*tdRFP*, *CerTNL15*, *CX3CR1*^+/−^ *EGFP*, or *SR101* labeled cells) affected by EAE – enables the direct identification of specific cellular origins of oxidative stress by examining colocalization of assembled NOX enzymes acquired via NAD(P)H–FLIM with cellular markers visualized by fluorescence imaging (Figures 4A,B).

We quantified the contribution of specific cell types to the total area of NOX enzymes activation in the CNS and found that the mean contribution of *LysM*^+^ phagocytes amounts to maximally 4.3%, a value comparable to that of neurons (Thy1^+^ cells, 4.7%). Whereas the contribution of neurons to the area of oxidative stress generation is, as expected, similar at peak EAE and during the remission phase, *LysM*^+^ phagocytes proved to be a major source of massive ROS production only during peak EAE but not during the remission, due to their low frequency within the CNS in this phase. Only few perivascular macrophages could be detected (Figure 4A, bottom panel) in line with the results of our FACS analysis in Figure 1A (5% of the cells are tdRFP expressing) and complementary to Figure 2A, in which no LysM tdRFP cell infiltration is shown after EAE recovery. LysM tdRFP cells can are located only in closest proximity to or within blood vessels (marked by white lines in Figure 4A) and are, even then, only partially activated (Figure 4A, bottom panel right image).

The main contribution to ROS production after recovery in EAE is associated with *CX3CR1*^+^ cells, i.e., 26%, and astrocytes (SR101), i.e., 45% (Figure 4B). Thus, microglia and astrocytes together contribute over 70% of the oxidative stress generation in remission of EAE. Since only in half of the microglia EGFP is detectable by intravital microscopy (heterozygous breeding) and not the entire astrocyte population takes up SR101 [i.e., only half of GFAP^+^ cells, namely GFAP^low^ (8)], the frequencies of microglia and astrocytes may well be underestimated, and we expect an even higher effective contribution of these cell types to oxidative stress.

## Discussion

It is widely accepted that during chronic neuroinflammation, both in the human disease MS and in its murine model EAE, oxidative stress plays a major role in demyelination and neuronal damage (6–8). Most of the findings reported in EAE studies resulted from investigations during the peak of inflammation (17). After peak of disease, in C57BL/6 mice immunized with MOG_35–55_ peptide (7), clinical signs can resolve spontaneously (10, 17) or the mice enter a chronic phase with persistent paralysis (1). This pattern resembles features of the human disease MS, since in its relapsing-remitting phase (RRMS) symptoms are normally self-limiting and patients spontaneously recover.

Despite our knowledge about the dynamic processes of the acute immune response in the CNS, information about the reaction of the tissue after the inflammatory attack is very limited. The processes determining how and where new lesions occur are difficult to study in human tissue as the majority of the samples derives either from very early (biopsies) or very late (post-mortem) lesions. How lesions resolve, how progression takes place, and other characteristics of the later phases of the disease are not well understood (3, 18–20). The alterations of the immune system in MS lesions are obvious, but how this impacts the function of the CNS tissue is almost unknown. Only few inflammatory animal models focus on remyelination as a tissue response, e.g., studies using models of inflammatory cortical demyelination (18, 19).

In this study, we addressed the question of tissue damage regarding oxidative stress as a major detrimental factor for the cells within CNS tissue and consecutive ongoing subclinical neuronal dysfunction after clinical remission of EAE signs. We observed that astrocytes and microglia are shifted toward an activated phenotype, showing both changes in morphology and, even more striking, a high level of activated NADPH oxidases correlating with persisting neuronal elevated calcium levels without evident morphological alterations. Hence, the consequences of sustained neuronal calcium levels after EAE recovery in contrast to peak of EAE are not clear yet (16). Could this be a reason for long-term neuronal damage leading to a progressive disease course in humans?

The role and fate of microglia/macrophages during neuroinflammation is still not well understood (10, 20). From BrdU studies, it was concluded that although microglia enter the cell cycle during acute inflammation, they return to quiescence following remission (21). Furthermore, gene expression analysis of microglia and macrophages suggested that after EAE recovery, these cells return toward homeostasis (10). In contrast, our data demonstrate that at least a subpopulation of microglia is not quiescent and still retains their activated function during remission, as demonstrated by their ameboid morphology and enhanced NOX activity in our model.

The results of our present study indicate that even after remission of the clinical signs, astroglial scars represent areas of ongoing tissue damage, even in the absence of peripheral immune cells. Our findings support the idea of a “trapped” ongoing CNS inflammation as a mechanism of chronic progression in MS (2). At this phase of the disease, astrocytes and microglia alone are a source of persistent oxidative stress locally correlating with ongoing subclinical neuronal dysfunction, as measured by pathologically increased calcium levels in *CerTN L15* × *LysM tdRFP* mice.

Our model provides a useful tool to further investigate the “tissue memory” of neuroinflammatory processes, in order to better understand mechanisms of chronicity and disease progression in MS.

## Materials and Methods

### Two-Photon Laser-Scanning Microscopy

Both fluorescence intensity and FLIM experiments were performed using a specialized two-photon laser-scanning microscope based on a commercial scan head (TriMScope, LaVision BioTec, Bielefeld, Germany). The detection of the fluorescence signals was accomplished either with photomultiplier tubes in the ranges 460 ± 30, 525 ± 25, 593 ± 20 nm or with a 16-channel parallelized TCSPC detector (FLIM-X_16_, LaVision BioTec, Bielefeld, Germany) in the range 460 ± 30 nm. The excitation of NADH and NADPH was performed at 760 nm (detection at 460 ± 30 nm), of Cerulean (detection at 460 ± 30 nm), SR101 (detection at 593 ± 20 nm), and EGFP (detection at 525 ± 25 nm) at 850 or 880 nm, and of tdRFP at 1110 nm (detection at 593 ± 20 nm).

For both intensity and fluorescence lifetime imaging, we used an average maximum laser power of 8 mW to avoid photodamage. The experimental parameters for FLIM were 160 ps histogram bin [for NAD(P)H–FLIM] and 80 ps histogram bin (for FRET–FLIM) and maximum acquisition time for a 512 × 512 image was 5 s to record a fluorescence decay stack. The time-window in which the fluorescence decays were acquired was set to 9 ns.

### Data Analysis

Three-dimensional intravital images acquired within the brain stem of *CX3CR1*^+/−^ *EGFP* mice, either healthy or affected by EAE, at peak and in the remission phase of the disease, were segmented using image analysis software (Imaris, BitPlane, UK). The 3D surfaces of the segmented cells (50–200 cells per condition) were transferred to Fiji/ImageJ and six orthogonal projections were generated for each cell. For each projection of each cell, the Fourier coefficients, describing the sphericity vs. ramification of the cell, were calculated (custom plug-in available in Fiji developed by Zoltan Cseresnyes). All Fourier coefficients corresponding to a single cell were merged following a linear algebraic combination to describe all ramifications of the cell (cell processes). The rank of the Fourier coefficients describes increasingly complex cellular ramifications: whereas the first Fourier coefficient corresponds to the first spherical approximation of the cell, the next coefficients describe increasingly longer processes.

Fluorescence lifetime imaging data analysis was performed using self-written software, as previously described (12, 14). The time-resolved fluorescence signal of NAD(P)H, as acquired by the TCSPC device, was approximated with a bi-exponential decay function (Eq. 1):

$$I_{\text{NAD}\left(\text{P}\right)\text{H}}(t)=\varepsilon +a_{1}\times e^{-\text{t}/\tau_{1}}+a_{2}\times e^{-\text{t}/\tau_{2}}$$

with ɛ the background, the 1-indexed term of the sum representing the fluorescence decay of free NADH and NADPH and the 2-indexed term representing the fluorescence decay of enzyme-bound NADH and NADPH. The fluorescence lifetime τ_1_ [free NAD(P)H] is 400–450 ps, while the fluorescence lifetime τ_2_ of NAD(P)H bound to metabolic enzymes has an average of ~2000 ps. If bound to NADPH oxidases (NOX1–4, DUOX1, 2), NADPH shows a typical fluorescence lifetime of 3650 ps, independent of cell type and even of species, since we repeatedly measured this value in various cell types of humans, mice, and even plants (*Nicotiana tabacum*). We focused all through the manuscript on the fluorescence lifetime τ_2_ of the enzyme bound NAD(P)H.

As previously described, we define the area of neuronal dysfunction as the area of free neuronal calcium exceeding a concentration of 1 μM (14). The neuronal calcium concentration was measured *in vivo* using FLIM, in mice expressing the FRET-based calcium biosensor TN L15 in Thy1^+^ cells. Thereby, the time-resolved fluorescence decay of the donor in the FRET construct (Cerulean) was also approximated by a biexponential function (Eq. 2):

$$I_{\text{Cerulean}}(t)=\varepsilon +a_{1}\times e^{-\text{t}/\tau_{1}}+a_{2}\times e^{-\text{t}/\tau_{2}}$$

with *ɛ* the background, the 1-indexed term representing the fluorescence decay of the FRET-quenched donor and the 2-indexed term representing the fluorescence decay of the unquenched donor. Here, we focused on the ratio *a*_1_/(*a*_1_ + *a*_2_) of the relative concentrations of the FRET-quenched *a*_1_ and unquenched Cerulean *a*_2_, and, using our previously published calibration curve, we determined the absolute calcium concentration within neurons.

Statistical analysis and graphical presentation was carried out with GraphPad Prism 4 (Graphpad Software, USA) and OriginPro (OriginLab, USA). Results are shown as mean values from analyzed data per mouse, in addition the mean ± SD summarize collective data from performed experiments.

### Mice

All mice used were on a C57BL/6 background. The *CerTN L15* × *LysM tdRFP* mouse expresses a FRET-based calcium biosensor consisting of Cerulean (donor) and Citrine (acceptor) bound to troponin C, a calcium-sensitive protein present in certain subsets of neurons (22). Additionally, tdRFP is expressed in LysM^+^ cells. The *CX3CR1*^+/−^ *EGFP* mouse was used to detect microglia (Table 1).

### EAE Induction

Experimental autoimmune encephalomyelitis was induced as previously described. Briefly, mice were immunized subcutaneously with 150 μg of MOG_35–55_ (Pepceuticals, UK) emulsified in CFA (BD Difco, Germany) and received 200 ng pertussis toxin (PTx, List Biological Laboratories, Inc.) intraperitoneally at the time of immunization and 48 h later. Intravital multi-photon microscopy was performed at different stages of the disease, i.e., peak (3–7 days after appearance of first clinical symptoms) and remission (after decline of clinical symptoms to a score ≤0.5). Mice were randomly picked for analysis. Detailed information about the performed EAE runs and individual scores of the mice are listed in Table 1. Mice at peak of disease were part of a previous study of Mossakowski et al. (8) and serve as a reference in this study. We did not included animals that never got sick and at onset analyzed animals.

### Preparation of the Brain Stem Window for Intravital Imaging

As previously described, the brain stem was exposed by carefully removing the musculature above the dorsal neck area and removing the dura mater between the first cervical vertebra and the occipital skull bone. The head was inclined for access to deeper brainstem regions and the brain stem superfused with isotonic Ringer solution. Anesthesia depth was controlled by continuous CO_2_ measurements of exhaled gas and recorded with a CI-240 Microcapnograph (Columbus Instruments, USA) and by an Einthoven three-lead electrocardiogram (ECG). In order to avoid strong breathing artifacts in the brainstem of anesthetized mice, the ECG signal was correlated to the respiration rate and used as an external trigger for the image acquisition software, which controls the hardware of the microscope setup. Thus, each fluorescence stack was recorded at the same respiration state of the mouse and also in the same tissue region. Animal experiments were approved by the appropriate state committees for animal welfare (G0081/10, LAGeSo – Landesamt für Gesundheit und Soziales) and were performed in accordance with current guidelines and regulations.

### FACS Analysis

To isolate cells from the whole brain and spinal cord of *LysM tdRFP* mice, the tissue was homogenized after PBS perfusion, and a percoll gradient was performed according to standard protocols with 25 and 75% stock istotonic percoll (GE Helthcare) and HBSS. Cells were blocked with antibodies to Fcγ receptors (DRFZ, clone 2.4G2) to avoid non-specific staining, and were subsequently stained with FITC-labeled PerCP-labled rat anti-CD45 (BioLegend) or Cy5- (DRFZ), APC- or Pacific Blue™ (BioLegend)-labeled rat anti-CD11b, in some experiments fixable Viability Dye eFluor^®^780 (eBioscience), anti-CX3CR1 APC and anti-CD3 Brilliant Violet™ (both BioLegend) were used according to standard procedures, followed by fixation using 4% Paraformaldehyde (Electron Microscopy Science) for 10 min. FACS analysis was performed on a LSR Fortessa (BD).

## Author Contributions

DB, HR, RN, and RG designed and performed research and analyzed data. DB, HR, RN, and RL wrote the manuscript. HR and RN initiated, organized, and supervised the project. HR and AH provided expertise in mouse handling and intravital imaging and performed EAE experiments (G0081/10). ZC developed algorithms and analyzed data.

## Conflict of Interest Statement

The authors declare that the research was conducted in the absence of any commercial or financial relationships that could be construed as a potential conflict of interest.
